# Identification of inhibitory immune checkpoints and relevant regulatory pathways in breast cancer stem cells

**DOI:** 10.1002/cam4.3902

**Published:** 2021-05-01

**Authors:** Haojun Shi, Yisi Yang

**Affiliations:** ^1^ School of Life Sciences Fudan University Shanghai China; ^2^ Graduate School of Asia‐Pacific Studies Waseda University Tokyo Japan

**Keywords:** breast cancer, cancer stem cells, immune checkpoints, immunotherapy

## Abstract

Immune checkpoint blockade (ICB) has become one of the most promising approaches to activating antitumor immunity. However, only a small subset of patients with breast cancer benefit from ICB treatment. To improve the therapeutic effect in the clinic, precision immunotherapy is proposed to accurately eliminate cancer stem cells that contribute to local recurrence or metastasis, but currently little is known about their immunological properties. In this study, breast cancer–specific datasets in The Cancer Genome Atlas were collected and analyzed by using multiple open‐access web servers. We found that the immunophenotype of breast cancer was characterized by a hypoactive immune microenvironment and a low response to immune checkpoint blockade. The innate immune checkpoint CD200 and the adaptive immune checkpoint CD276, respectively, exhibited a strong correlation with basal/stem gene signature and invasiveness gene signature, both of which represent breast cancer stem cells. Wnt, TGF‐β, and Hedgehog signaling, which are recognized as stemness‐related pathways, showed a significant association with the expression of CD200 and CD276, suggesting cancer stem cell–specific immune checkpoints could be downregulated by inhibiting these pathways. Of note, levels of CD200 and CD276 expression were higher in TGF‐β dominant breast cancer than in other immune types of breast cancer. We also identified gene signatures that represent Wnt, TGF‐β, and Hedgehog signaling‐related CD200 and CD276 expression in breast cancer stem cells. For the luminal A subtype, the patient group with a high level of these gene signatures plus a low infiltration of CD8^+^ T cells, or dendritic cells, or M1 macrophages had poor overall survival. Our study suggested that CD200 and CD276 are candidate inhibitory immune checkpoints in breast cancer stem cells, which are potentially regulated by Wnt, TGF‐β, and Hedgehog signaling. Synergistic inhibition of these stemness‐related pathways may improve the efficacy of ICB treatment targeting breast cancer stem cells in precision immunotherapy.

## Graphical abstract

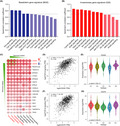

## 1 INTRODUCTION

The concept and practice of modern immunotherapy have brought a new era for cancer treatment modalities previously limited to surgery, radiotherapy, chemotherapy, and hormone or targeted therapy. Immune checkpoint blockade (ICB) as a first‐line or second‐line therapy has been shown to provide a survival advantage in the clinical management of melanoma,^1^, ^2^ Hodgkin's lymphoma,^3^, ^4^ lung cancer,^5^, ^6^ and colon cancer.^7^ In the setting of breast cancer, the first breakthrough for clinically applied ICB was reported in the IMpassion130 trial where atezolizumab plus nab‐paclitaxel prolonged progression‐free survival among patients with metastatic triple‐negative breast cancer.^8^ Unfortunately, the benefit of anti‐PD‐L1 antibody is restricted to patients whose tumors have ≥1% PD‐L1‐positive tumor‐infiltrating lymphocytes.^8^ Despite progress in a subtype of breast cancer with limited therapeutic options, the current limitations of immunotherapy in the treatment of breast cancer are inevitably highlighted.

A comprehensive comparison of the immune microenvironment among common types of cancer shows that lower responsiveness to ICB in breast cancer is partially explained by its immunological quiescence characterized by a low mutational burden and a paucity of tumor‐infiltrating lymphocytes.^9^, ^10^ On the other hand, the precise elimination of cancer's source—cancer stem cells—by immunotherapy has not been implemented to improve the therapeutic effect in the clinic. Cancer stem cells constitute a rare population of malignant cells endowed with self‐renewal and tumor‐initiating capabilities,^11^, ^12^ and, more importantly, may function as an indispensable reservoir for tumor relapse following radical surgery and adjuvant therapy.^13^, ^14^ Since stem cells are immune privileged in specialized niches,^15^ long‐living cancer stem cells may escape from immunosurveillance while more differentiated cancer cells are constantly eliminated. The recent report that adaptive immune privilege emerges from melanoma‐initiating cells^16^ has demonstrated the immunological properties of cancer stem cells. It is also possible that breast cancer stem cells may drive local recurrence or distant metastasis as deadly seeds through immune checkpoint‐mediated immune evasion. However, currently little is known about cancer stem cell immunology.

Here, we used the datasets of breast invasive carcinoma from The Cancer Genome Atlas (TCGA) to identify cancer stem cell–specific inhibitory immune checkpoints in breast cancer. Since attenuation of the stemness of breast cancer cells may downregulate the expression of their immune checkpoints, we also determined the regulatory pathways associated with stemness and immune checkpoints, and constructed gene signatures, which represent Wnt, TGF‐β, and Hedgehog signaling‐related CD200 and CD276 expression in breast cancer stem cells, to predict the prognosis of patients with breast cancer. In‐depth knowledge of cancer stem cell immunology in breast cancer will be helpful for the design of efficient immunotherapies targeting breast cancer stem cells to prevent and treat cancer recurrence and metastasis.

## 2 METHODS

### 2.1 Study cohorts

The datasets of patients were from TCGA, which were collected by multiple open‐access web servers for bioinformatic analysis, including The Cancer Immunome Atlas (TCIA), Gene Expression Profiling Interactive Analysis 2 (GEPIA2), The cBio Cancer Genomics Portal (cBioPortal), Tumour Immune Estimation Resource 2 (TIMER2), and TISIDB. TCIA includes bladder urothelial carcinoma (BCLA, *n* = 412), breast invasive carcinoma (BRCA, *n* = 1098), squamous cell carcinoma and endocervical adenocarcinoma (CESC, *n* = 307), colon adenocarcinoma (COAD, *n* = 462), glioblastoma multiforme (GBM, *n* = 604), head and neck squamous cell carcinoma (HNSC, *n* = 528), kidney chromophobe (KICH, *n* = 113), kidney renal clear cell carcinoma (KIRC, *n* = 537), liver hepatocellular carcinoma (LIHC, *n* = 377), lung adenocarcinoma (LUAD, *n* = 574), lung squamous cell carcinoma (LUSC, *n* = 504), ovarian serous cystadenocarcinoma (OV, *n* = 591), pancreatic adenocarcinoma (PAAD, *n* = 185), rectum adenocarcinoma (READ, *n* = 171), skin cutaneous melanoma (SKCM, *n* = 471), stomach adenocarcinoma (STAD, *n* = 443), thyroid carcinoma (THCA, *n* = 507), and uterine corpus endometrial carcinoma (UCEC, *n* = 560). GEPIA2 includes 1085 cases of BRCA. cBioPortal (Firehouse legacy) includes 1108 cases of BRCA. TIMER2 includes 1100 cases of BRCA. TIMER2 includes 1100 cases of BRCA. TISIDB includes 1082 cases of BRCA. Although the number of BRCA cases from TCGA was updated in some open‐access web servers, it did not affect the analysis results. Because all data that this study used were from publicly available datasets TCGA and CTRP, no ethical approval was required to seek.

### 2.2 TICA analysis

The Cancer Immunome Atlas (TCIA) is a database describing the intratumoral landscapes and the cancer antigenomes from 20 solid cancers on the basis of the TCGA datasets 8243 samples.^17^ A deconvolution approach CIBERSORT was used to identify fractions of immune subpopulations in the different types of tumor tissues.^18^ A comprehensive view of total immune infiltration across multiple cancer types was provided, and cell fraction of 22 subpopulations of tumor‐infiltrating lymphocytes was estimated. In addition, single‐sample gene set enrichment analysis (ssGSEA) was performed to decompose cellular profiles from bulk RNA sequencing data for individual samples.^19^, ^20^ The association was represented by Z‐score normalized NES (Z(NES)) with a threshold of 1, and an immune cell type was considered enriched in a patient if the false discovery rate (q‐value) was <0.25. Moreover, neo‐antigens were predicted by mutations that reside within exons and do not span exon borders. The tumors were classified into three groups according to the median mutation number.

### 2.3 GEPIA2 analysis

Gene Expression Profiling Interactive Analysis 2 (GEPIA2) is an enhanced online tool for 9736 tumor samples across 33 cancer types and 726 adjacent normal tissues from TCGA and Genotype‐Tissue Expression (GTEx).^21^, ^22^ The raw data in the web server were beforehand recomputed by the UCSC Xena project based on a uniform pipeline to avoid incompatibility. In our study, only TCGA datasets were used to ensure no bias caused by sampling. When performing comparison of the selected immune checkpoint genes across multiple cancer types, we choose log2(TPM+1)‐transformed expression data for plotting. The density of color in each block represents the median expression value of a gene in a given tissue, normalized by the maximum median expression value across all blocks. Meanwhile, a survival map across cancer types and multiple Kaplan–Meier plots were obtained to show the results based on gene expression levels. Overall and disease‐free survival analyses of multiple genes were performed with a significant level of 0.05 and a cut‐off threshold of 50% for both low and high expression groups. Moreover, Spearman's correlation coefficient between given genes or signatures was obtained by using pair‐wise gene expression correlation analysis. The signatures of stemness‐related pathways were obtained from the Molecular Signatures Database (MSigDB) v7.2.

### 2.4 CellMinerCDB analysis

CellMinerCDB is an open‐access tool for integrating genomics and pharmacogenomics analyses of cancer cell lines.^23^ In our study, the datasets from the Cancer Therapeutics Response Portal (CTRP) ^24^ were chosen to analyze the mutual correlation of the selected immune checkpoint genes in 37 breast cancer cell lines. A heat map was generated to exhibit the correlation coefficient between those immune checkpoint genes.

### 2.5 cBioPortal analysis

The cBio Cancer Genomics Portal (cBioPortal) is a web‐based resource to explore multidimensional cancer genomics data including somatic mutations, copy number alterations (CNAs), DNA methylation, mRNA expression, protein abundance, and phosphoprotein abundance.^25^, ^26^ In our study, mutations and CNAs of breast cancer from TCGA (Firehose Legacy) were used to display a graphical summary of genomic alterations and identify mutually exclusive or co‐occurrent events in the selected immune checkpoint genes. An odds ratio was calculated to indicate the likelihood, and a value over 2 means a tendency toward co‐occurrence. The results with q‐value <0.05 were considered statistically significant.

### 2.6 TIMER2 analysis

Tumour Immune Estimation Resource 2 (TIMER2) provides a robust estimation of the immune infiltrate population for TCGA.^27^ With the function of tumor purity adjustment, TIMER2.0 can minimize the influence of immune cells in the microenvironment when the expression of a certain gene in cancer cells is analyzed. In our study, purity‐corrected partial Spearman's correlation analysis was used to recognize the coexpression pattern of the selected immune checkpoint genes in breast cancer. We then examined how immunocytes and immune checkpoint‐related signatures are associated with patient survival on Kaplan–Meier curves.

### 2.7 TISIDB analysis

TISIDB is an integrated repository portal of the TCGA datasets to investigate the interaction between tumor and immune system.^28^ Spearman's correlation between the abundance of tumor‐infiltration lymphocytes and expression of a given gene was explored to examine which kinds of cells may be regulated by the select gene in our study. For each cancer type, the relative abundance of tumor‐infiltration lymphocytes was deduced by gene set variation analysis (GSVA) based on 28 immunocyte signatures.^17^ Additionally, the distribution of the select immune checkpoint genes across immune subtypes of breast cancer was checked and displayed in violin plots.^29^

### 2.8 Statistical analysis

Comparison between two groups was performed by Student's t‐test. Association between two groups was analyzed by Spearman's correlation analysis. Survival analysis was performed based on the Kaplan–Meier analysis and log‐rank test. Overall and disease‐free survival was defined as the time between the date of surgery and date of death or the date of the last follow‐up. A *p*‐value <0.05 was considered statistically significant.

## 3 RESULTS

### 3.1 Profiling of immune infiltrates and inhibitory immune checkpoints across multiple cancer types

**FIGURE 1:**
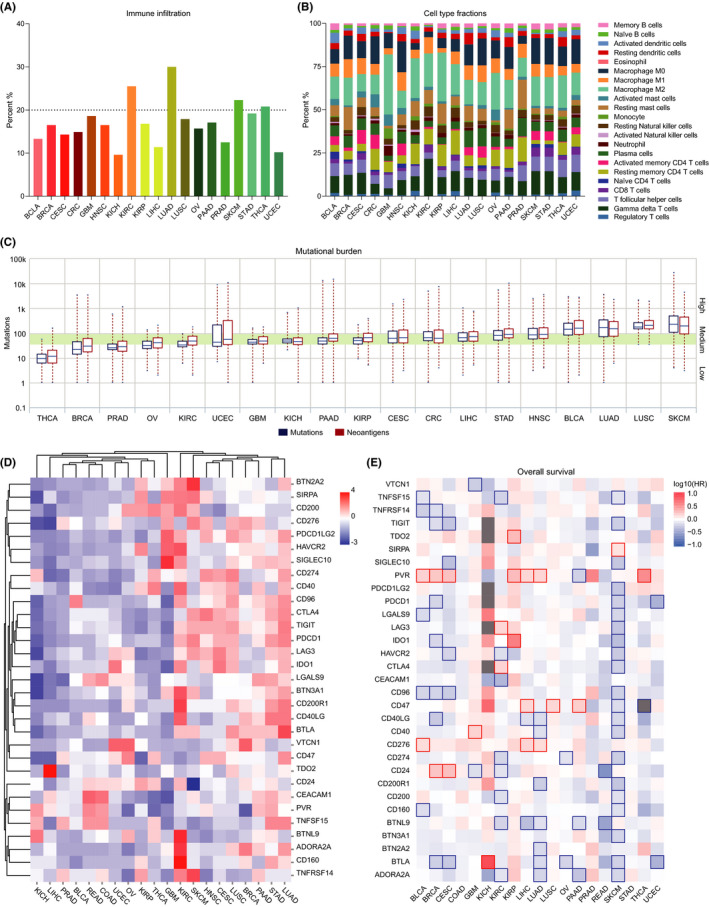
Profiling of immunophenotypes across multiple cancer types. (A) Infiltration of total immunocytes in solid tumors. (B) Proportion of various immune subpopulations in solid cancers. (C) Variance of mutations and neoantigens in solid tumors. Tumors are sorted into high, medium, and low burden group according to the mutation number. (D) Expression profiles of the selected immune checkpoints in solid tumors. (E) Contribution of the selected immune checkpoints to overall survival in solid tumors. The red blocks indicate that a high level of a given gene is unfavorable to survival while the blue blocks indicate that a high level of a given gene is favorable to survival. The Cox proportional hazard ratio is included in the survival plots

Immune microenvironment in tumor tissues can be stratified on the basis of immune infiltrates and mutational burden.^9^ To have a comprehensive understanding of immune heterogeneity across multiple cancer types, we used TCIA to mine the TCGA data from 20 solid tumors.^17^ COAD and READ were combined as a single entity, CRC. All cancer types were analyzed by CIBERSORT, a deconvolution approach, to characterize cell composition of immune microenvironment from their gene expression profiles.^18^ LUAD, KIRC, SKCM, and THCA were the top four types enriched with various immunocyte gene signatures (Figure 1A). Their estimated infiltration rate of immunocytes all exceeded 20%, and even one of them reached 30%. On the contrary, only KICH had less than 10% of tumor‐infiltrating immunocytes.

Analysis of cell type fractions across multiple cancer types by a leukocyte gene signature matrix which distinguishes 22 immunocyte phenotypes showed that CD8^+^ T cells did not account for more than 5% in each cancer type and that macrophages constituted about one third of tumor‐infiltrating immunocytes (Figure 1B). In fact, innate immunity including dendritic cells, natural killer cells, monocytes, macrophages, and neutrophils took up more than half of the immune microenvironment in each cancer type, especially GBM. Meanwhile, we performed single‐sample gene set enrichment analysis (ssGSEA) to estimate heterogeneity of immune infiltrates within individual cancer entities.^19^, ^20^ Macrophages and neutrophils were enriched in almost half of the patients with various cancer types while memory B cells, NK‐T cells, eosinophils, and mast cells generally had a small proportion, less than 20% (Figure S1). Since immunogenic neoantigens expressed by cancer cells can induce immune responses,^30^, ^31^ we observed the variance of mutational burden to estimate their immunogenicity. The 19 solid tumors were classified by quartiles into three groups: high mutational burden (HNSC, BCLA, LUAD, LUSC, and SKCM), intermediate mutational burden (STAD, LIHC, CRC, CESC, KIRP, PAAD, KICH, UCEC, and GBM), and low mutational burden (KIRC, OV, PRAD, BRCA, and THCA) (Figure 1C). Thus, a high level of heterogeneity in immune microenvironment spans across multiple cancer types.

Considering ICB is among the most promising approaches to activating therapeutic antitumor immunity, we selected 31 inhibitory immune checkpoints that were previously reported to play a role in tumorigenesis or tumor progression and observed their expression across multiple cancer types by using GEPIA2.^21^, ^22^ SKCM, HNSC, CESC, LUSC, STAD, LUAD, and PAAD expressed a higher level of the immune checkpoint gene signature while LIHC and KICH are in the cluster with the lowest level (Figure 1D). Besides, a few immune checkpoints, such as TDO2, PVR, IDO1, CD276, and CD24, were found negatively correlated with both overall and disease‐free survival of the patients while most of the checkpoints did not predict the prognosis of patients as expected (Figure 1E and Figure S2A). One of the explanations is that some checkpoints may be specific to certain subpopulations such as cancer stem cells, of which the information is covered up by the whole in bulk RNA sequencing data.

To estimate the responsiveness of the 19 solid tumors to ICB, we then classified their immunophenotypes on the basis of immune infiltrates, mutational burden, and immune checkpoint gene signature. SKCM, LUSC, BCLA, LUAD, and KIRC were clustered independently due to active immune response and/or a high level of immune checkpoint gene signature (Figure S2B), suggesting those tumors may be optimal for ICB treatment as reported.^32^ On the contrary, the cluster containing PRAD, KICH, LIHC, and UCEC was characterized by a hypoactive immune microenvironment and/or a low level of immune checkpoint gene signature, which implies a relatively low antitumor response rate after routine ICB treatment.

### 3.2 Identification of inhibitory immune checkpoints in breast cancer stem cells

Stratification of intertumor heterogeneity in breast cancer is routine during the course of diagnosis and treatment to achieve better clinical outcomes.^33^ However, insights from studies on cellular heterogeneity and plasticity raise the possibility that multiple tumor subclones with distinct molecular characterization may coexist within a tumor.^34^, ^35^, ^36^ That also means the bulk data might not provide an accurate reflection of the phenotype and genotype of specific subclones. To test whether the unrefined information of the 31 selected inhibitory immune checkpoints can reveal the clinical outcome of heterogeneous breast cancer, we compared the expression of these immune checkpoints in tumor tissues and normal tissues and evaluated the relationship among their expression, immune infiltration, and prognosis. The adaptive immune checkpoints BTNL9 and VTCN1 and the innate immune checkpoints CD200 and SIRPA were downregulated in breast tumor tissues while the metabolic immune checkpoints ADORA2A and TDO2 were upregulated in breast tumor tissues (Table S1). Survival analysis showed that despite no difference between expressions in normal and tumor tissues (Figure S3A and D) and no association with the infiltration of various immunocytes (Figure S3G and H), high expression of CD24 or PVR was significantly unfavorable for overall and disease‐free survival of patients with breast cancer (Figure S3B, C, E, and F). However, some immune checkpoints, such as BTLA, CD40LG, CD96, and IDO1, were unexpectedly associated with a good prognosis (Table S1). It is plausible that the contradiction between these results and the reported protumor functions of immune checkpoints may be caused by the fact that some immune checkpoints are only expressed in certain tumor subclones and the mistaken perception that unique expression of an immune checkpoint in a certain tumor subclone necessarily affects the immune responses to the whole tumor. These results also suggested that studies on immune checkpoints should take into consideration the tumor subclones to which they belong.

**FIGURE 2:**
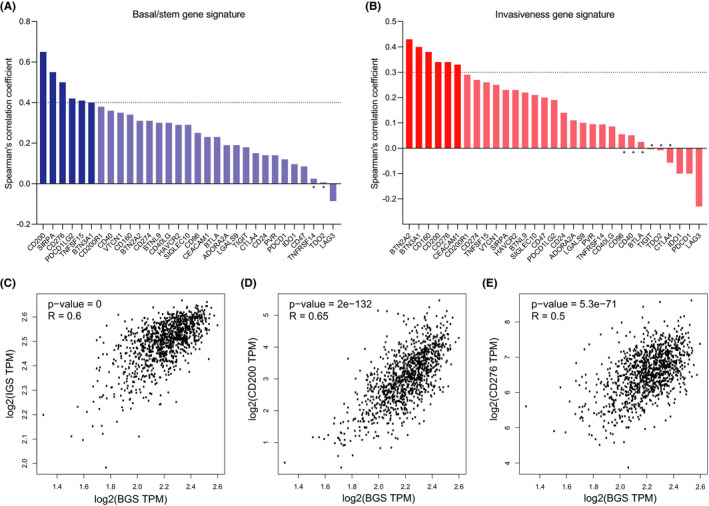
Potential relevance between the inhibitory immune checkpoints and breast cancer stem cells. (A) Correlation of the selected immune checkpoints with basal/stem gene signature (BGS) in breast cancer. The threshold of Spearman's correlation coefficient was set at 0.4. *, *p*‐value ≥0.05. (B) Correlation of the selected immune checkpoints with invasiveness gene signature (IGS) in breast cancer. The threshold of Spearman's correlation coefficient was set at 0.3. *, *p*‐value ≥0.05. (C) Scatter plot for correlation between BGS and IGS in breast cancer. (D) Scatter plot for correlation between CD200 expression and BGS in breast cancer. (E) Scatter plot for correlation between CD276 expression and BGS in breast cancer

Cancer stem cells at the apex of cell hierarchy are able to differentiate aberrantly into heterogeneous subclones of cancer cells.^37^ To identify inhibitory immune checkpoints in breast cancer stem cells, we evaluated the correlation between the selected immune checkpoints and two individual sets of gene signature representative of cancer stem cells. The basal/stem gene signature (BGS) is enriched in metastasis‐initiating cells of human breast cancer.^38^ The invasiveness gene signature (IGS) was generated through comparison of the gene expression profiles of CD44^+^ CD24^−/low^ tumor‐initiating cells and normal breast epithelium.^39^ As expected, BGS and IGS were correlated closely in breast cancer (Figure 2C), which indicates that high correlation with each of these two gene signatures can represent a strong correlation with breast cancer stem cells. Correlation analysis showed that the expression of CD200, SIPRA, CD276, PDCD1LG2, and TNFSF15 was found significantly correlated with IGS, respectively (Figure 2A, Figure S4A), while the expression of BTN2A2, BTN3A1, CD160, CD200, CD276, and CEACAM1 was found significantly correlated with BGS, respectively (Figure 2B, Figure S4B). Taken together, we considered CD200 and CD276, respectively, as potential innate and adaptive immune checkpoints in breast cancer stem cells (Figure 2D and E).

**FIGURE 3:**
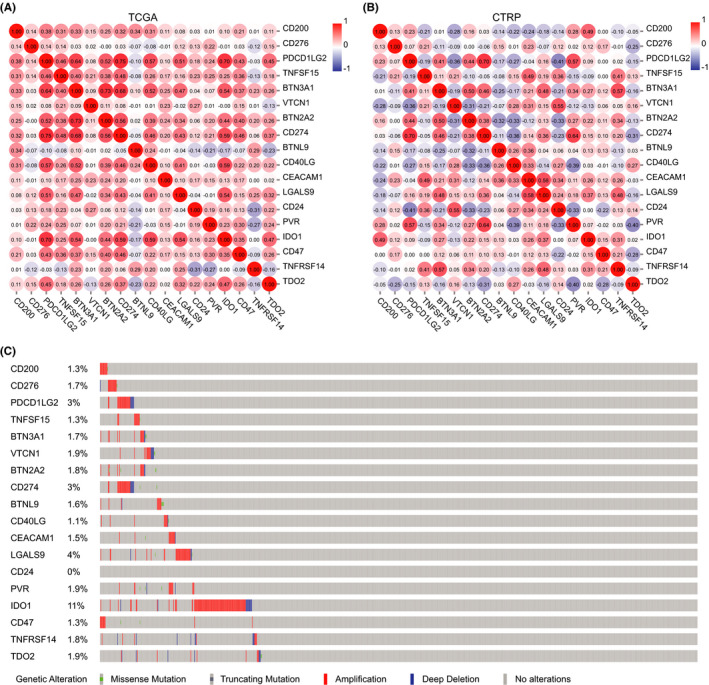
Mutual relevance of the inhibitory immune checkpoints in breast cancer. (A) Profile of correlation between the immune checkpoints in breast cancer based on the TCGA data. The value in the circle is Spearman's correlation coefficient. (B) Profile of correlation between the immune checkpoints in breast cancer cell lines based on the CTRP data. The value in the circle is Pearson's correlation coefficient. (C) Landscape of genetic alterations of the immune checkpoints in breast cancer

Next, we wondered whether a subpopulation of cancer cell stems have the dual capabilities of resistance to the innate and adaptive immune system through synchronous expression of CD200 and CD276. We adopted the “Tumour Purity Adjustment” function in TIMER2.0 when performing the correlation analysis,^27^ which reduces the bias caused by mixture with immunocytes during TCGA data processing. CD200 expression was found to have no strong correlation with the other immune checkpoints expressed by breast cancer cells, and neither was CD276 expression (Figure 3A, Figure S4C). In addition, we used CellMinerCDB to further prove this in 37 breast cancer cell lines.^23^ Consistently, no significant correlation was detected between CD200, or CD276 expression, and the other immune checkpoints (Figure 3B, Figure S4D), which suggested that expression of CD200 and CD276 may be independently regulated, and that heterogeneity may still exist in cancer stem cell population, particularly in the aspect of immune checkpoint. Of note, mutual exclusivity analysis on the basis of mutation and copy number data in the cBioPortal^25^, ^26^ revealed that CD200 exhibited significant co‐occurrence with CD47 (Figure 3C and Table 1), which implies that CD47 may disrupt phagocytic clearance of noncancer stem cells while CD200 is responsible for protecting cancer stem cells from phagocytes.

**TABLE 1:** Mutual exclusivity analysis of inhibitory immune checkpoints in breast cancer

A	B	Neither	A Not B	B Not A	Both	Log2 Odds Ratio	*p*‐Value	q‐Value	Tendency
PDCD1LG2	CD274	932	0	2	29	>3	<0.001	<0.001	Co‐occurrence
BTN3A1	BTN2A2	944	2	3	14	>3	<0.001	<0.001	Co‐occurrence
CEACAM1	PVR	942	3	7	11	>3	<0.001	<0.001	Co‐occurrence
CD200	CD47	946	4	4	9	>3	<0.001	<0.001	Co‐occurrence
TNFSF15	CD274	923	9	27	4	>3	<0.001	0.016	Co‐occurrence
TNFSF15	TNFRSF14	936	10	14	3	>3	0.001	0.026	Co‐occurrence
PDCD1LG2	BTN2A2	921	25	13	4	>3	0.001	0.026	Co‐occurrence
TNFSF15	PVR	935	10	15	3	>3	0.001	0.027	Co‐occurrence
BTN2A2	CD274	919	13	27	4	>3	0.002	0.027	Co‐occurrence
BTNL9	TDO2	933	12	15	3	>3	0.002	0.033	Co‐occurrence
BTN3A1	VTCN1	932	13	15	3	>3	0.003	0.034	Co‐occurrence
BTN3A1	PVR	932	13	15	3	>3	0.003	0.034	Co‐occurrence
VTCN1	PVR	930	15	15	3	>3	0.004	0.044	Co‐occurrence

### 3.3 Identification of regulatory pathways for cancer stem cell–specific immune checkpoints

**FIGURE 4:**
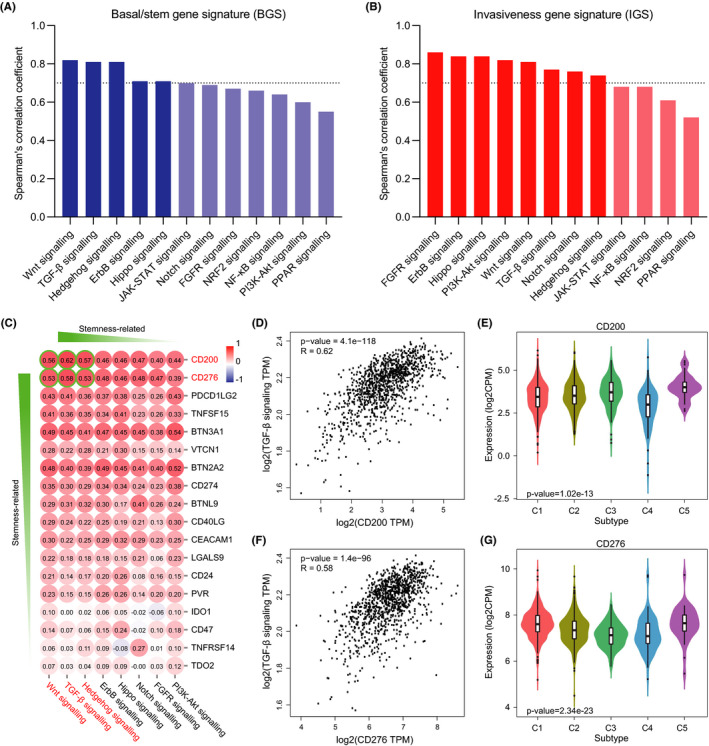
Potential relevance between the inhibitory immune checkpoints and stemness‐related pathways in breast cancer. (A) Correlation of BGS with stemness‐related pathways in breast cancer. The threshold of Spearman's correlation coefficient was set at 0.7. All *p*‐values were <0.001. (B) Correlation of IGS with stemness‐related pathways in breast cancer. The threshold of Spearman's correlation coefficient was set at 0.7. All *p*‐values were <0.001. (C) Profile of correlation between the immune checkpoints in breast cancer. The red font represents stemness related. The value in the circle is Spearman's correlation coefficient. (D) Scatter plot for correlation between CD200 expression and TGF‐β signaling in breast cancer. (E) Violin plots for CD200 expression in multiple immune types of breast cancer. C1, wound healing; C2, IFN‐γ dominant; C3, inflammatory; C4, lymphocyte depleted; C5, TGF‐β dominant. (F) Scatter plot for correlation between CD276 expression and TGF‐β signaling in breast cancer. (G) Violin plots for CD276 expression in multiple immune types of breast cancer

Inhibition of stemness‐related pathways has been reported not only to reduce the tumorigenic activity of cancer stem cells but also to impair expression of multiple immune checkpoints on cancer stem cells, including PD‐L1, TIM3, and CD24.^40^ It is also possible that blockade of stemness‐related pathways in breast cancer may impede immune checkpoint‐mediated immune evasion of cancer stem cells. To determine which pathways regulate breast cancer stem cells, we sorted the reported signalings according to their respective correlation with BGS and IGS. Correlation analysis showed that the pathway signatures, of which the correlation coefficient with BGS was over 0.7, were Wnt, TGF‐β, Hedgehog, ErbB, and Hippo signaling (Figure 4A). They were also among the signatures of which the correlation coefficient with IGS was over 0.7 (Figure 4B), indicating that these five signalings could be strongly associated with the regulation of breast cancer stem cells.

We then determined the exact pathway which controls the cancer stem cell–specific immune checkpoints CD200 and CD276. TGF‐β, Hedgehog, and Wnt signaling signatures were strongly associated with CD200 and CD276 expression, while Hippo and ErbB signatures had a relatively weak association with them (Figure 4C). That also means that cancer stem cells with high TGF‐β, Hedgehog, and Wnt signaling activation are able to display high levels of CD200 and CD276 expression, thus escaping from immunosurveillance. Correspondingly, noncancer stem cell–specific immune checkpoints had a poor association with the five stemness‐related pathways (Figure 4C). Of note, CD200 and CD276 expression had their respective higher correlation coefficient with TGF‐β signaling signature than with other signaling signatures (Figure 4C, D, and F). Using TISIBD, we also noticed that the levels of CD200 and CD276 expression were higher in the TGF‐β dominant subtype of breast cancer (Figure 4E and G) which was enriched with TGF‐β signature and lymphocytic infiltrate but suffered the worst overall survival^29^ than in other immune subtypes.

**FIGURE 5:**
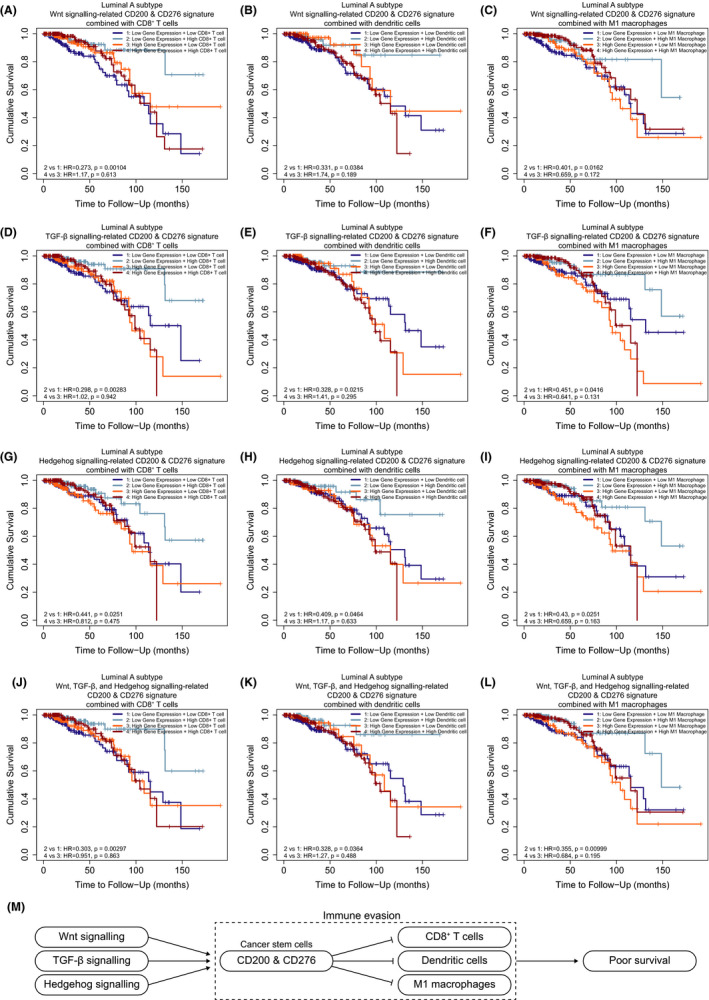
Prognostic value of cancer stem cell–specific immune checkpoint signature plus immune infiltration level in patients with luminal A subtype of breast cancer. (A–C) Kaplan–Meier curves for overall survival split by level of Wnt signaling‐related CD200 and CD276 signature and infiltration level of CD8^+^ T cells, dendritic cells, or M1 macrophages in breast cancer. (D–F) Kaplan–Meier curves for overall survival split by level of TGF‐β signaling‐related CD200 and CD276 signature and infiltration level of CD8^+^ T cells, dendritic cells, or M1 macrophages in breast cancer. (G–I) Kaplan–Meier curves for overall survival split by level of Hedgehog signaling‐related CD200 and CD276 signature and infiltration level of CD8^+^ T cells, dendritic cells, or M1 macrophages in breast cancer. (J–L) Kaplan–Meier curves for overall survival split by level of Wnt, TGF‐β, and Hedgehog signaling‐related CD200 and CD276 signature and infiltration level of CD8^+^ T cells, or dendritic cells, or M1 macrophages in breast cancer. (M) A hypothetical model of cancer stem cell–specific immune checkpoints. Activation of Wnt, TGF‐β, and Hedgehog signaling upregulates CD200 and CD276 expression in breast cancer stem cells and thus impedes the tumoricidal function of CD8^+^ T cells, dendritic cells, and M1 macrophages. Immune evasion mediated by cancer stem cell–specific immune checkpoints leads to poor survival in patients with breast cancer

As breast cancers can be classified into basal/stem cell–enriched and luminal/differentiated cell‐enriched clusters,^38^ we used logistic regression to screen out gene signatures (Table S2) which indicate Wnt, TGF‐β, and Hedgehog signaling‐related CD200 and CD276 in breast cancer stem cells. We evaluated the predictive power of these gene signatures in a fivefold cross‐validation procedure. After 100 repetitions, the average accuracy of Wnt, TGF‐β, and Hedgehog signaling‐related CD200/CD276 signatures was 91.42%, 92.68%, and 86.73%, respectively. To further clarify the clinical significance and the interaction of cancer stem cell–specific immune checkpoints with the immune microenvironment, we estimated their prognostic value in breast cancer. For the luminal A subtype, any patient group with either a high level of these gene signatures or a low infiltration of CD8^+^ T cells, or dendritic cells, or M1 macrophages had a high risk of poor overall survival (Figure 5A–L). However, such observations appeared inconsistent in basal, HER2‐enriched, and luminal B subtypes (Figure S5–S8). That suggested the infiltration of CD8^+^ T cells, dendritic cells, or M1 macrophages in breast cancer can signify prolonged patient survival, and that the expression of CD200 and CD276 in cancer stem cells could impair the tumoricidal function of CD8^+^ T cells, dendritic cells, and M1 macrophages. Therefore, Wnt, TGF‐β, and Hedgehog signaling are potentially the main pathways regulating the inhibitory immune checkpoints CD200 and CD276 in breast cancer stem cells.

## 4 DISCUSSION

Precision immunotherapy has been proposed to ensure that the potency of the immune system is oriented toward the source of tumorigenesis, recurrence, and metastasis, namely cancer stem cells. Successful delivery of precision immunotherapy to patients with breast cancer requires a comprehensive understanding of heterogeneous immune microenvironment and cancer stem cell immunology. In this study, we identified CD200 and CD276, respectively, as candidate innate and adaptive immune checkpoints in breast cancer stem cells. Wnt, TGF‐β, and Hedgehog signaling were found strongly associated with cancer stem cell–specific immune checkpoints. Moreover, we identified gene signatures that represent Wnt, TGF‐β, and Hedgehog signaling‐related CD200 and CD276 expression in breast cancer stem cells. The patient group with a high level of these gene signatures plus a low infiltration of CD8^+^ T cells, or dendritic cells, or M1 macrophages had poor overall survival.

The functional characteristics of a cancer stem cell have been defined as the capacities to self‐renew and to differentiate into heterogeneous lineages of cancer cells which constitute a tumor.^41^ Unfortunately, little is known about the immunological properties of cancer stem cells. The question whether or not the tumor‐initiating capacity of cancer stem cells depends on their immune privilege rose from the inconsistency between the frequencies of melanoma‐initiating cells in mouse models with distinct immune responses. One melanoma‐initiating cell of 46,700 melanoma cells was estimated to form a tumor in NOD/SCID mice lacking T and B cells while the frequency of tumor‐initiating cell was one of nine melanoma cells in NSG mice lacking T, B, and NK cells.^42^ It can be partially explained by the assumption that cancer stem cells may be much less vulnerable to immunosurveillance than more differentiated cancer cells. NK cell–deficient NSG mice appear to neither eliminate cancer stem cells nor more differentiated cancer cells which also become capable of initiating tumors. Mechanistically, ABCB5^+^ melanoma‐initiating cells preferentially blunted IL‐2‐dependent T‐cell activation and induced infiltration of regulatory T cell through low levels of MHC class I and tumor‐associated antigens and high levels of the costimulatory molecules B7.2 and PD‐1.^43^ Meanwhile, squamous cell carcinoma stem cells were reported to selectively express CD80 to suppress cytotoxic T cell–mediated tumoricidal activity, resulting in refractoriness to adoptive T‐cell transfer therapy.^16^ In the context of breast cancer, CD44^high^ CD24^low^ HER2^low^ cancer stem cells exhibited resistance to antibody‐dependent cell‐mediated cytotoxicity induced by trastuzumab and NK cells.^44^ Following the principle, our study revealed CD200 and CD276 as candidate immune checkpoints in breast cancer stem cells. Consistently, CD200 was found to be expressed in breast cancer cell line MDA‐MB‐231, along with cancer stem cell marker CD44^+^ CD24^−^.^45^ Enrichment of CD276 expression increased stemness of breast cancer cell lines by activation of the MAPK kinase pathway.^46^ However, more convincing evidence of CD200 and CD276 expressed in human breast cancer stem cells has not been provided. Our study showed a strong association of CD200 and CD276 expression with cancer stem cells, which implies unique immune features of cancer stem cells and increases the possibility of utilizing immunotherapy to eliminate them.

Because cancer stem cells have been defined as a rare population that are able to seed secondary tumors, it has also been proposed that cancer stem cells could be responsible for distant metastasis. Multicolor lineage tracing in mouse models demonstrated that polyclonal lung metastases arise from collective dissemination of cell clusters containing keratin 14‐expressing tumor‐initiating cells.^47^, ^48^ Yet, the molecular mechanism has not been clarified, which keeps metastasis‐initiating cells alive when they travel along blood vessels, invade distant organs, keep quiescent in niches, and finally overtake host tissue. The finding that metastatic cancer cells downregulated their cell surface innate immune sensors during latency periods ^49^ suggested the possibility of resistance to immune surveillance in metastatic stem cells and the feasibility of immune checkpoints as biomarkers of metastatic stem cells. Coincidentally, cell surface proteome profiling revealed CD200 as one of the markers specifically upregulated on metastatic breast cancer primary explants.^50^ Expression of CD200 was also significantly upregulated in bone, lung, and liver metastatic lesions of breast cancer.^51^ Meanwhile, the positive correlation between CD276 mRNA expression and the number of circulating tumor cells was observed in blood specimens of gastric cancer patients.^52^ It is reasonable to suppose that CD200 and CD276 could also be surface markers of metastatic stem cells in circulation. If the hypothesis that immune evasion is one of the fundamental properties in metastatic stem cells is confirmed, cancer stem cell–specific immune checkpoints will have its advantages to be used to measure metastatic burden or capture circulating tumor cells in a blood‐based liquid biopsy^53^ because circulating tumor cells in patients with epithelial cancers might express mesenchymal rather than epithelial markers due to epithelial‐to‐mesenchymal transition.^54^

Long‐term clinical observations found that estrogen receptor (ER)‐positive breast cancer maintains a significant risk of relapse even after more than 10 years of follow‐up,^55^ which suggests that ER‐positive breast cancer has a propensity for metastatic latency. Our results suggested that cancer stem cell–specific CD200 and CD276 might inhibit the surveillance of innate and adaptive immune system in luminal A breast cancer, thus contributing to poor survival. Consequently, it can be inferred that CD200‐positive or CD276‐positive cancer stem cells may orchestrate metastatic latency through immune evasion. ICB targeting these metastatic stem cells which stay in a quiescent state before micrometastasis formation appears a sound strategy to prevent and treat cancer metastasis. Furthermore, since stemness and immune evasion are closely intertwined with each other in cancer, disruption of stemness‐related pathways may also overcome immune evasion of cancer stem cells by suppressing the expression of inhibitory immune checkpoints. In breast cancer, β‐catenin transcriptionally upregulates N‐glycosyltransferase STT3, which consequently mediates glycosylation of PD‐L1 and prevents PD‐L1 from ubiquitin/proteasome‐mediated degradation.^56^ Meanwhile, low doses of doxorubicin reduced Akt‐activated β‐catenin levels, thus downregulating diverse immune checkpoints in leukemia stem cells, such as PD‐L1, TIM3, and CD24.^40^ Pharmacological inhibition of an RNA *N* ^6^‐methyladenosine demethylase dramatically impaired self‐renewal of leukemia stem cells and, simultaneously, attenuated immune response by suppressing immune checkpoint genes including LILRB4.^57^ Anti‐TGF‐β antibodies rendered TGF‐β‐responsive cancer stem cells sensitive to adoptive T‐cell transfer treatment, of which the efficacy could be hindered by CD80/CTLA4‐mediated immunosuppression.^16^ Therefore, synergistic blockade of cancer stem cell–specific immune checkpoints and relevant stemness‐related pathways is hypothesized to enhance the efficacy of immunotherapy targeting metastatic stem cells.

Pan‐cancer analysis of immune characteristics identified the TGF‐β dominant subtype that had a high lymphocytic infiltrate but the least favorable outcome.^29^ The contradiction implies the existence of immunosuppression in the TGF‐β dominant subtype, consistent with our finding that two cancer stem cell–specific immune checkpoints were higher in the TGF‐β dominant subtype than in other immune subtypes. In preclinical studies, TGF‐β signaling has displayed its effects on the immune microenvironment in multiple ways. For example, increased TGF‐β enhances antigen‐induced PD‐1 expression on T cells in a SMAD3‐dependent manner,^58^ drives exclusion of cytotoxic T cells, and impairs acquisition of a Th1 effector phenotype in a cancer metastasis model which has a limited response to anti‐PD‐L1 therapy.^59^ Moreover, TGF‐β‐mediated stromal remodeling attenuates tumor response to PD‐L1 blockade by restriction of CD8^+^ T‐cell infiltration.^60^ Given the potential synergistic effect of TGF‐β signaling blockade and ICB, a phase I trial of anti‐PD‐L1/TGFβRII fusion protein M7824 (NCT03579472) is being performed in patients with metastatic triple‐negative breast cancer. In this study, the strong association of TGF‐β signaling with the cancer stem cell–specific immune checkpoints implies that inhibition of TGF‐β signaling may impede CD200‐ or CD276‐mediated immune evasion of breast cancer stem cells, which has rarely been described before. The recognition of this mechanism will expand the application scenarios of TGF‐β signaling blockade in immunotherapy.

The current study still has some limitations which need to be addressed in the future. First, a single‐cell transcriptomic map of human breast cancer based on a large number of samples will help to more accurately and efficiently figure out inhibitory immune checkpoints and their regulatory pathways in breast cancer stem cells. Second, our results were derived from bioinformatic analysis, which needs further experiments in patient‐derived xenograft or mouse models to confirm them. Finally, the therapeutic effect of a combination of ICB and relevant signaling blockade on local recurrence and metastasis requires clinical trials to verify. Despite these limitations, identification of cancer stem cell–specific immune checkpoints and relevant regulatory pathways is anticipated to promote the development of cancer stem cell immunology.

In summary, our study revealed that CD200 and CD276 may act as cancer stem cell–specific immune checkpoints to mediate immune resistance in breast cancer. Synergistic inhibition of stemness‐related pathways including Wnt, TGF‐β, and Hedgehog signaling may improve the efficacy of ICB treatment targeting CD200 or CD276 in breast cancer stem cells. This combination treatment will provide a novel and efficient strategy for precision immunotherapy.

## CONFLICT OF INTEREST

The authors declare no competing interests.

## ETHICAL APPROVAL

Because all data this study used were from publicly available datasets TCGA and CTRP, no ethical approval was needed.

## Supporting information

Fig S1

Fig S2

Fig S3

Fig S4

Fig S5

Fig S6

Fig S7

Fig S8

Table S1

Table S2

## Data Availability

This study used publicly available datasets from The Cancer Genome Atlas (TCGA) and Cancer Therapeutics Response Portal (CTRP).
