# Supplementary figures and images for: Identification of inhibitory immune checkpoints and relevant regulatory pathways in breast cancer stem cells
Source: Cancer Med. 2021 May 1;10(11):3794–807. doi: 10.1002/cam4.3902 (PMC8178503; doi:10.1002/cam4.3902)

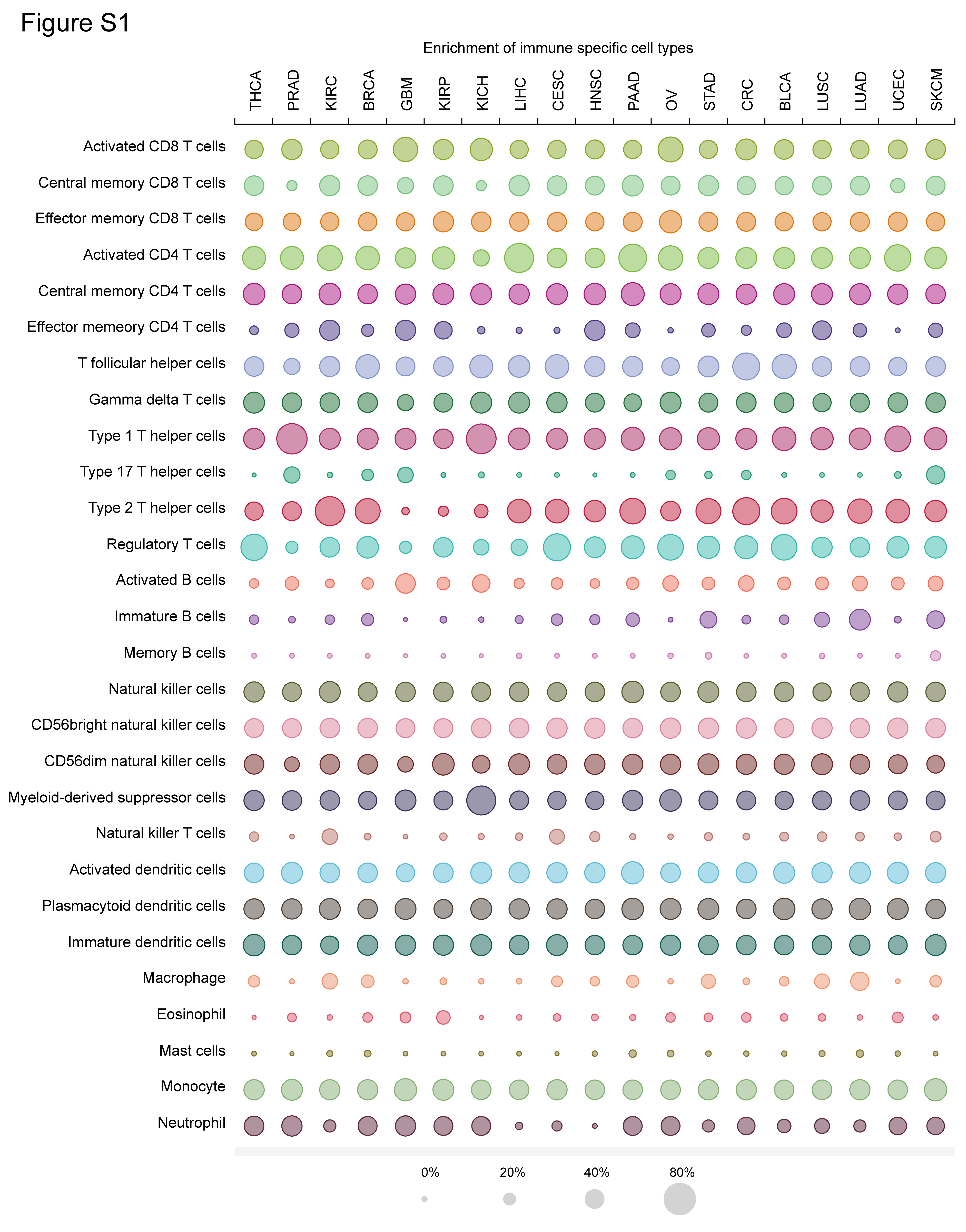

Supplement: Supplementary file 1 — Fig S1 [file CAM4-10-3794-s007.tif]

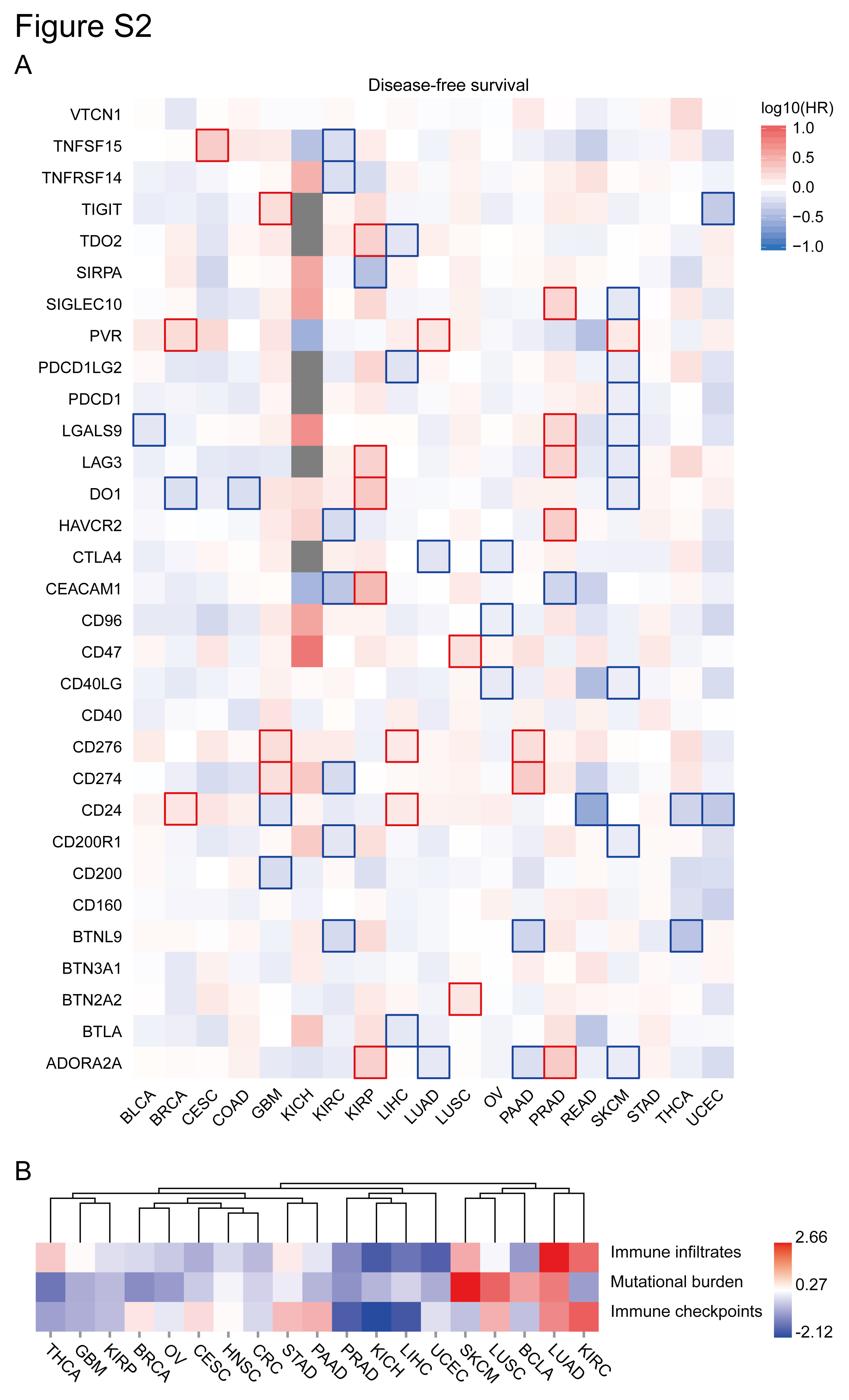

Supplement: Supplementary file 2 — Fig S2 [file CAM4-10-3794-s003.tif]

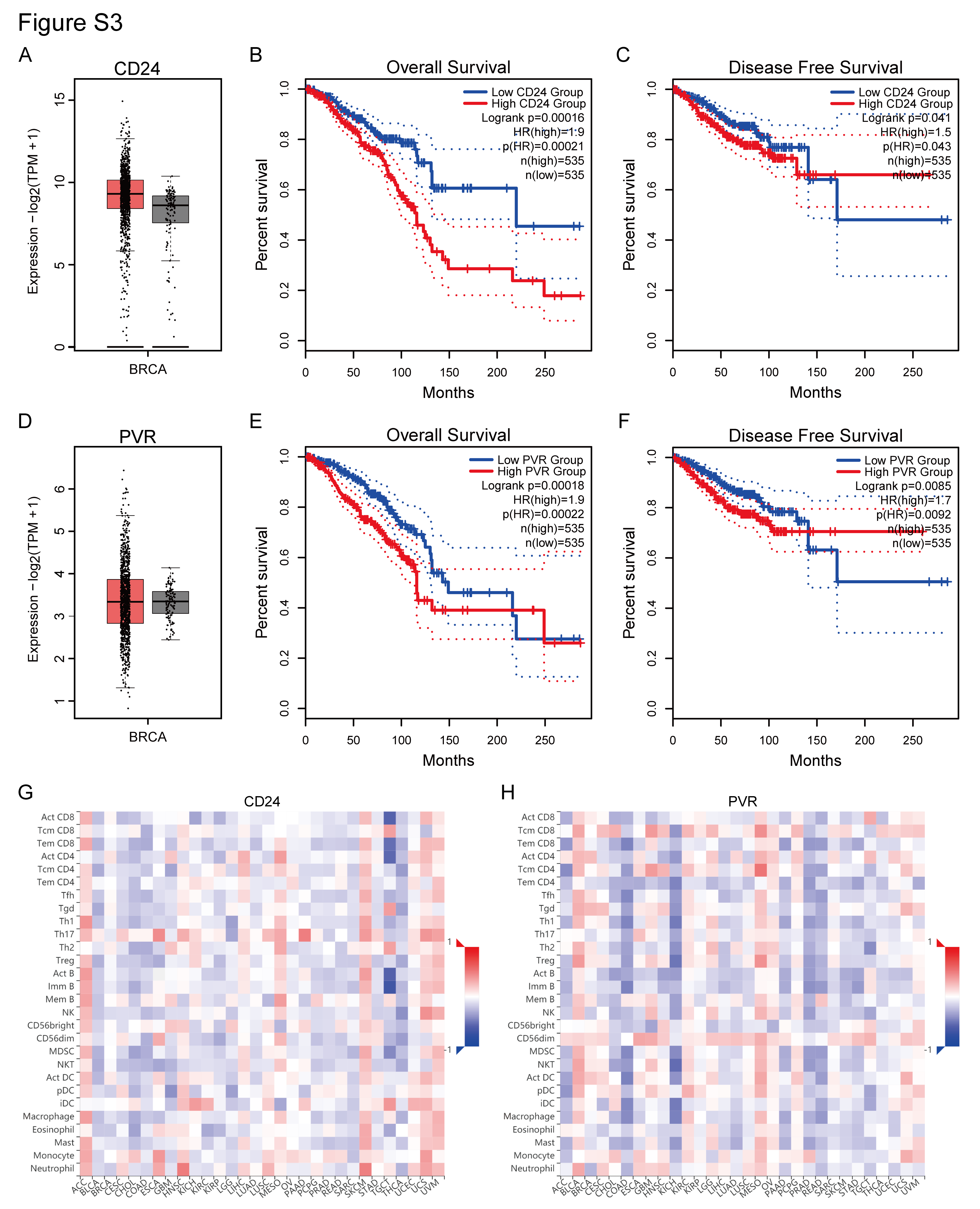

Supplement: Supplementary file 3 — Fig S3 [file CAM4-10-3794-s008.tif]

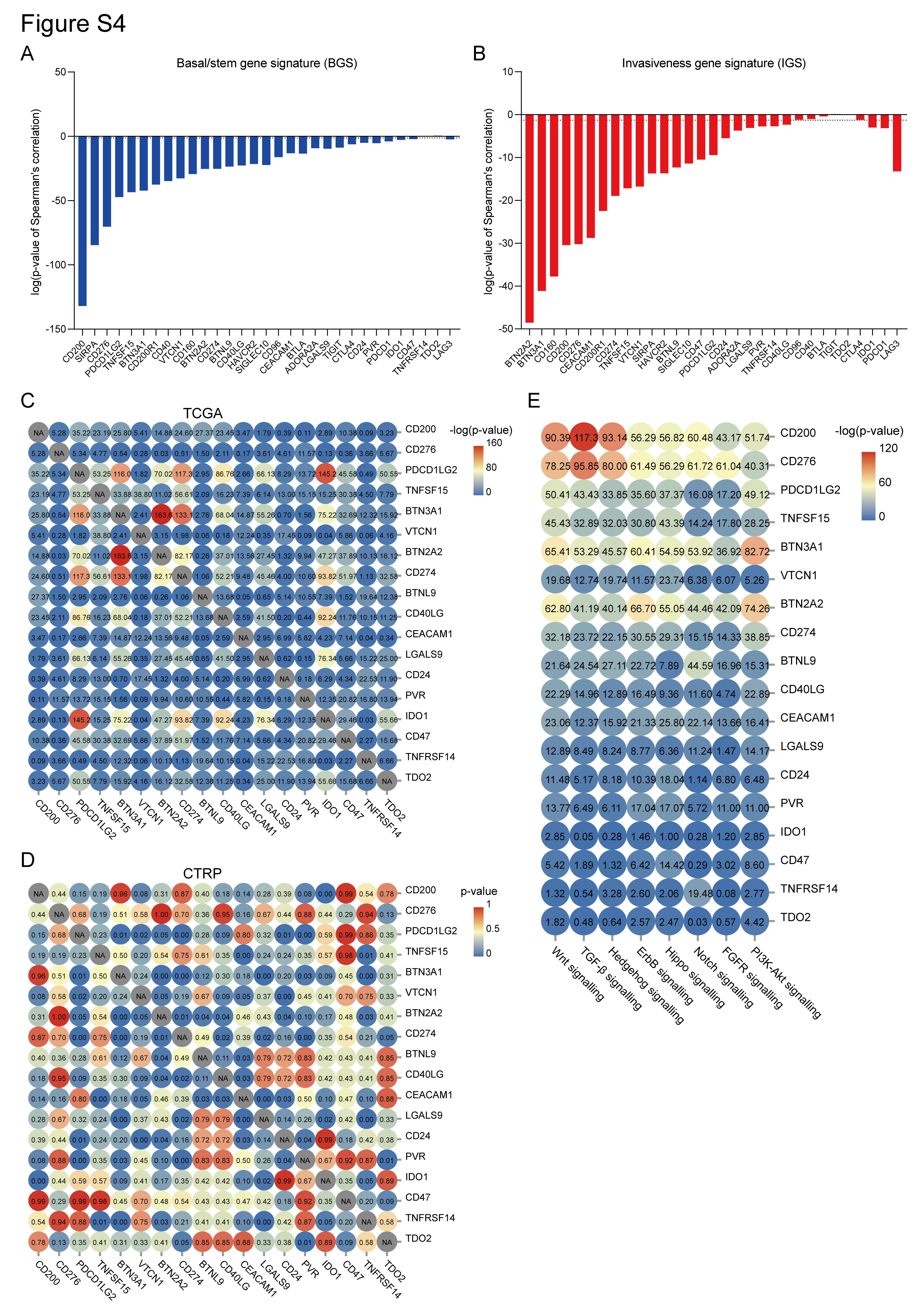

Supplement: Supplementary file 4 — Fig S4 [file CAM4-10-3794-s002.tif]

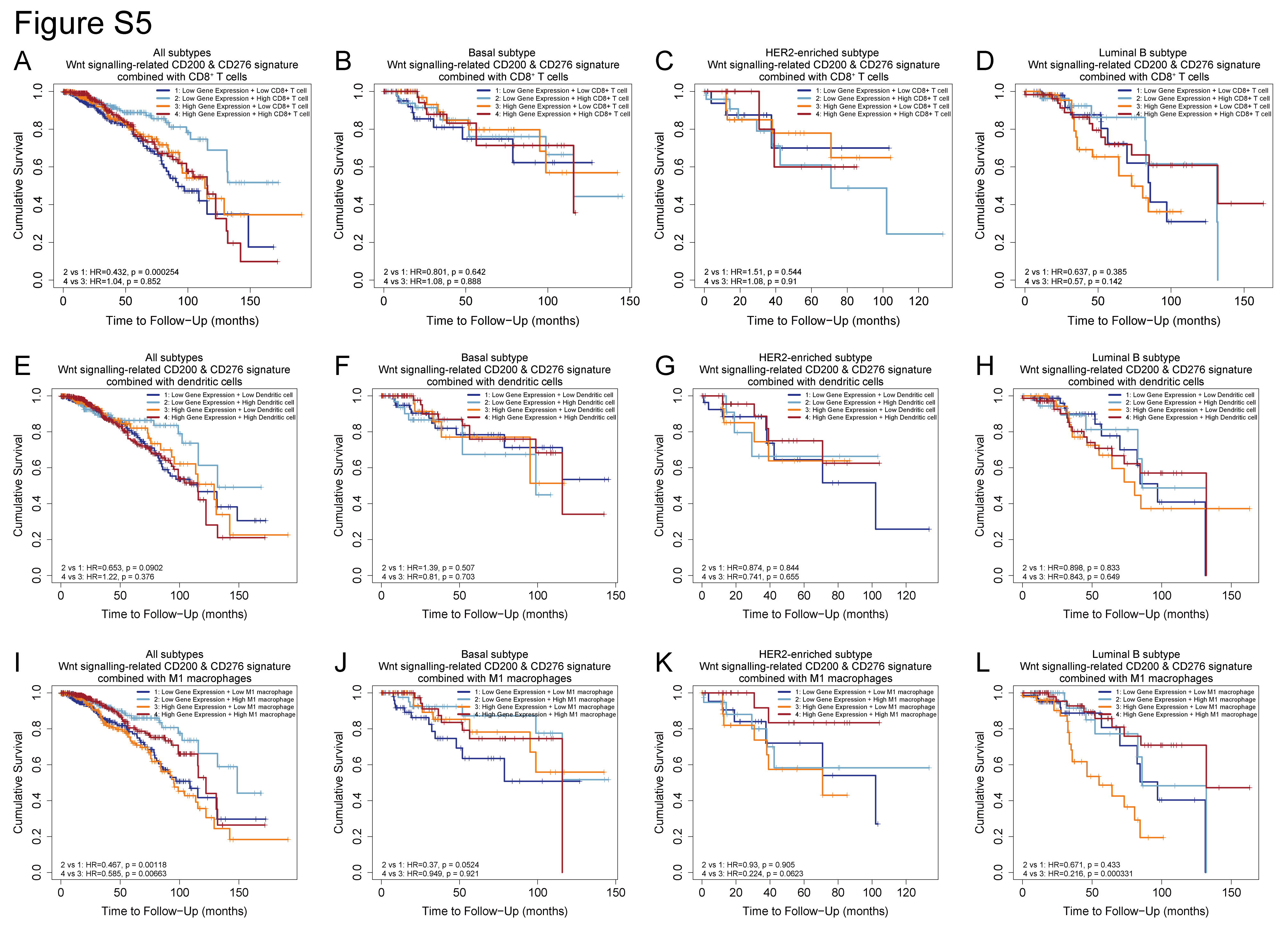

Supplement: Supplementary file 5 — Fig S5 [file CAM4-10-3794-s006.tif]

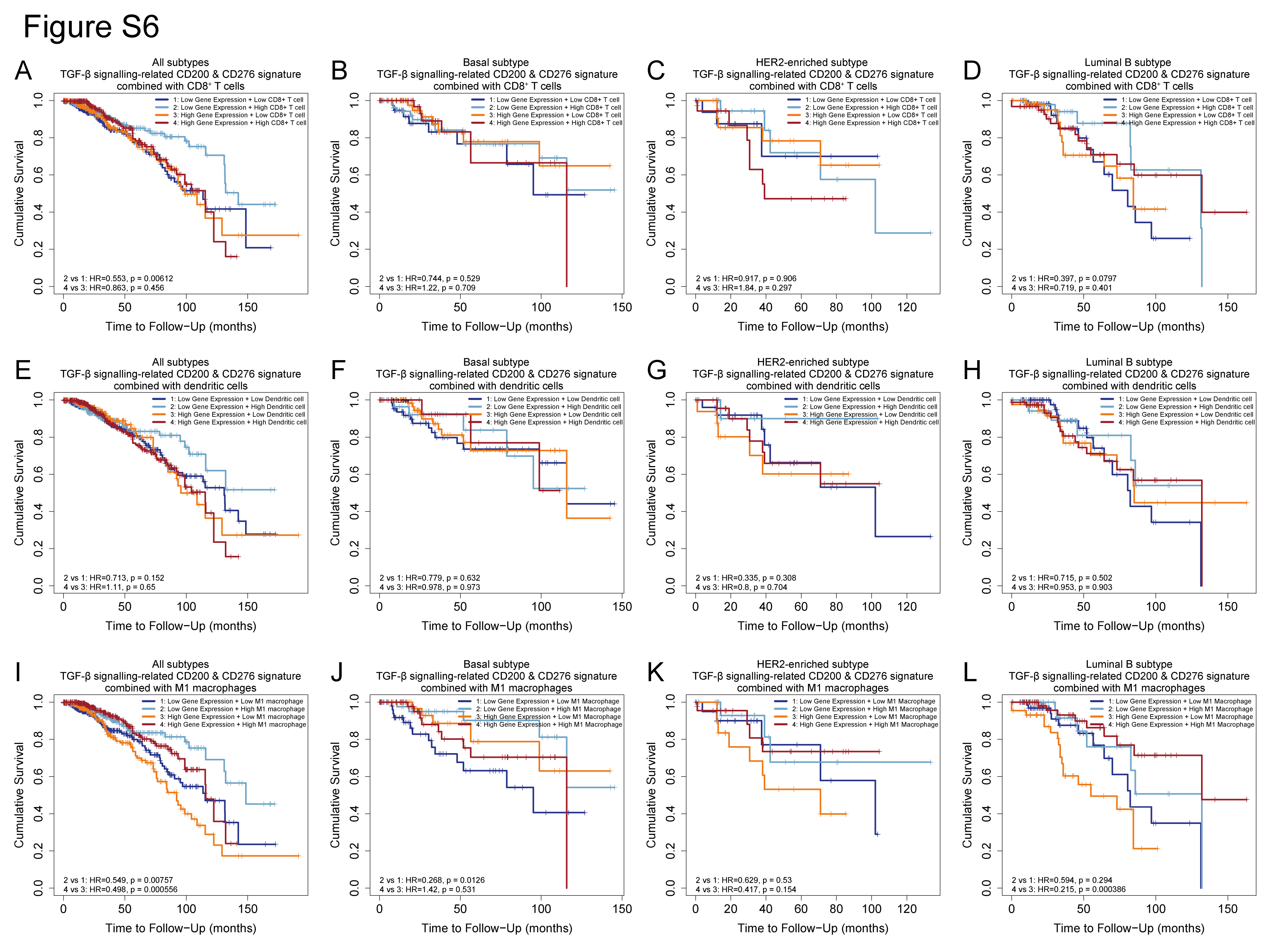

Supplement: Supplementary file 6 — Fig S6 [file CAM4-10-3794-s005.tif]

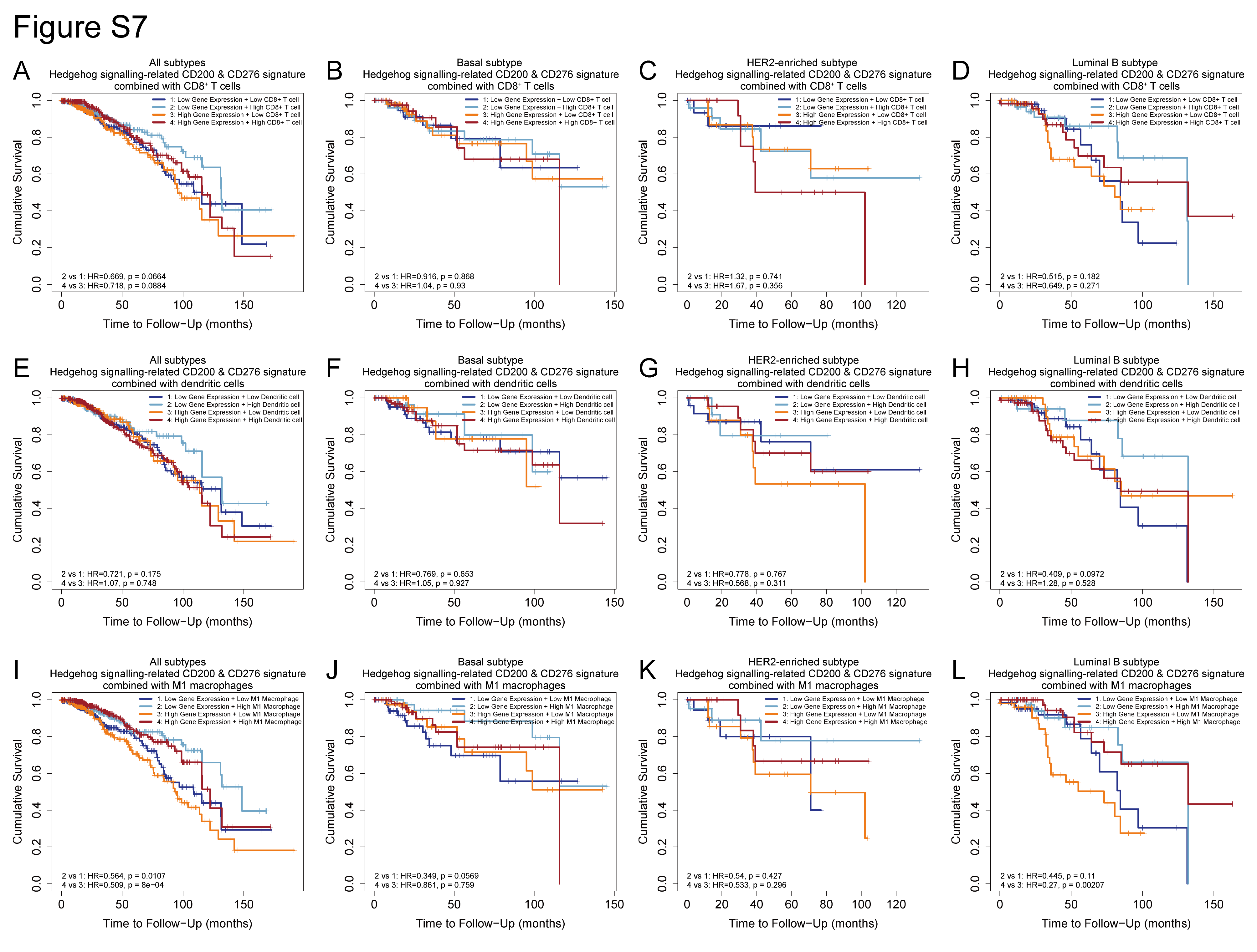

Supplement: Supplementary file 7 — Fig S7 [file CAM4-10-3794-s004.tif]

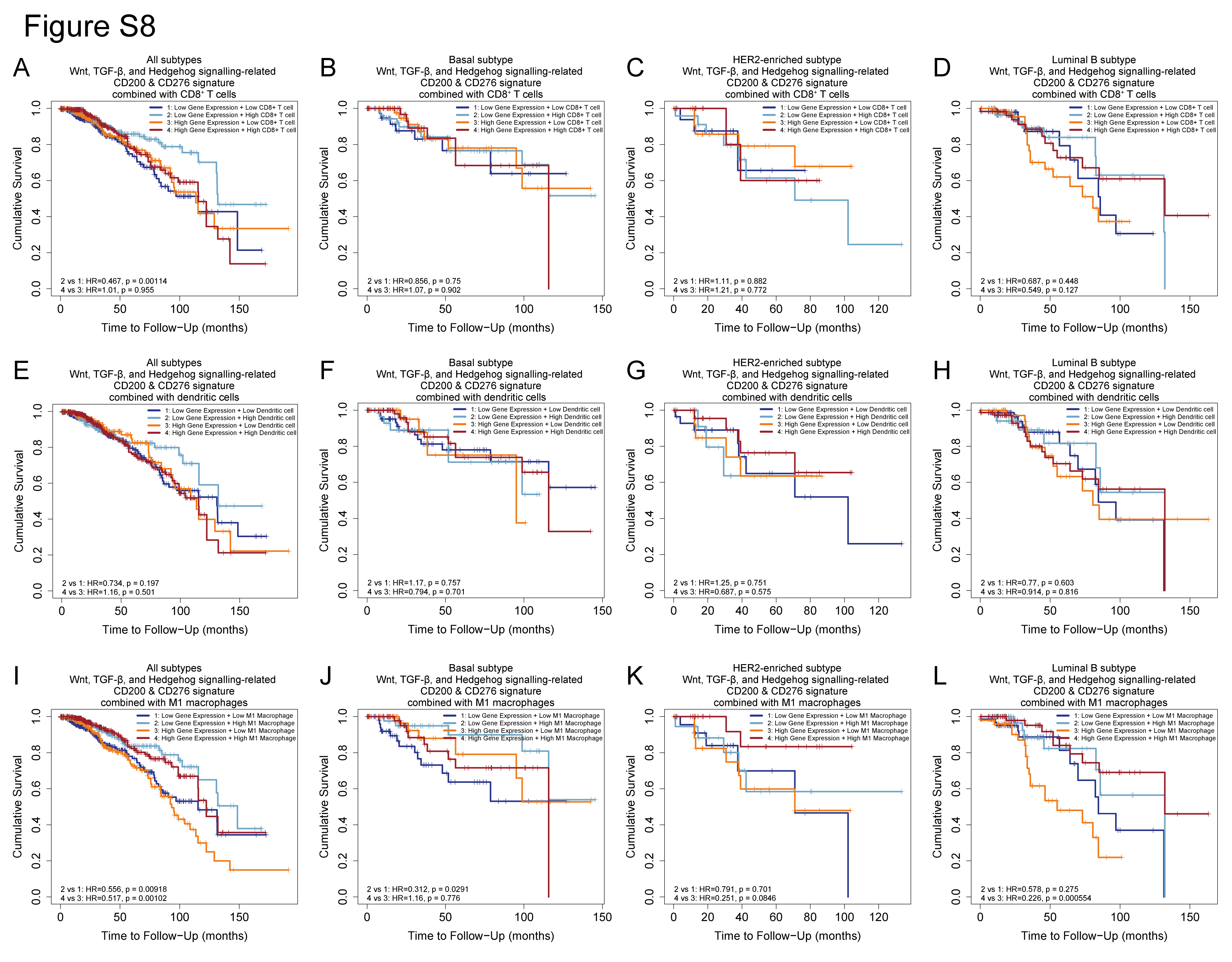

Supplement: Supplementary file 8 — Fig S8 [file CAM4-10-3794-s001.tif]
