# Supplementary material for: Identification of inhibitory immune checkpoints and relevant regulatory pathways in breast cancer stem cells
Source: Cancer Med. 2021 May 1;10(11):3794–807. doi: 10.1002/cam4.3902 (PMC8178503; doi:10.1002/cam4.3902)
Supplement: Supplementary file 9 — Table S1 [file CAM4-10-3794-s009.docx]

**Table S1 Prognostic value of inhibitory immune checkpoints in breast cancer**

| **Checkpoint** | **Cell type specificity (APCs/Tumour cells)** | **Expression (Tumour *vs.* Normal)** | **OS** | |  | **DFS** | | |
| --- | --- | --- | --- | --- | --- | --- | --- | --- |
|  |  |  | **HR** | **p-Value** |  | **HR** | **p-Value** |  |
| ADORA2A | No | Up | 0.88 | 0.42 |  | 1.1 | 0.75 |  |
| BTLA | No | Ns | 0.58 | 0.0011 |  | 0.81 | 0.27 |  |
| BTN2A2 | Yes | Ns | 0.73 | 0.063 |  | 0.69 | 0.052 |  |
| BTN3A1 | Yes | Ns | 0.81 | 0.2 |  | 0.72 | 0.095 |  |
| BTNL9 | Yes | Down | 1.1 | 0.65 |  | 1.1 | 0.67 |  |
| CD160 | No | Ns | 0.75 | 0.08 |  | 0.86 | 0.42 |  |
| CD200 | Yes | Down | 1 | 0.89 |  | 0.92 | 0.67 |  |
| CD200R1 | No | Ns | 0.82 | 0.23 |  | 0.87 | 0.45 |  |
| CD24 | Yes | Ns | 1.9 | 0.00031 |  | 1.5 | 0.043 |  |
| CD274 | Yes | Ns | 0.85 | 0.3 |  | 0.83 | 0.33 |  |
| CD276 | Yes | Ns | 1.1 | 0.46 |  | 0.94 | 0.74 |  |
| CD40 | No | Ns | 0.81 | 0.21 |  | 0.91 | 0.61 |  |
| CD40LG | Yes | Ns | 0.56 | 0.00064 |  | 0.73 | 0.1 |  |
| CD47 | Yes | Ns | 0.96 | 0.8 |  | 0.86 | 0.42 |  |
| CD96 | No | Ns | 0.61 | 0.0093 |  | 0.74 | 0.12 |  |
| CEACAM1 | Yes | Ns | 0.92 | 0.62 |  | 0.75 | 0.13 |  |
| CTLA4 | No | Ns | 0.75 | 0.09 |  | 0.9 | 0.58 |  |
| HAVCR2 | No | Ns | 0.85 | 0.33 |  | 0.97 | 0.88 |  |
| IDO1 | Yes | Ns | 0.65 | 0.01 |  | 0.64 | 0.023 |  |
| LAG3 | No | Ns | 1 | 0.94 |  | 1 | 0.87 |  |
| LGALS9 | Yes | Ns | 0.83 | 0.27 |  | 0.82 | 0.3 |  |
| PDCD1 | No | Ns | 0.65 | 0.01 |  | 0.86 | 0.42 |  |
| PDCD1LG2 | Yes | Ns | 0.91 | 0.58 |  | 0.7 | 0.066 |  |
| PVR | Yes | Ns | 1.9 | 0.00022 |  | 1.7 | 0.0092 |  |
| SIGLEC10 | No | Ns | 0.88 | 0.43 |  | 1 | 0.87 |  |
| SIRPA | No | Down | 0.91 | 0.56 |  | 1.3 | 0.19 |  |
| TDO2 | Yes | Up | 1.2 | 0.38 |  | 1.2 | 0.29 |  |
| TIGIT | No | Ns | 0.66 | 0.013 |  | 0.8 | 0.24 |  |
| TNFRSF14 | Yes | Ns | 0.63 | 0.006 |  | 0.73 | 0.098 |  |
| TNFSF15 | Yes | Ns | 1.1 | 0.44 |  | 1.1 | 0.69 |  |
| VTCN1 | Yes | Down | 1 | 0.99 |  | 0.7 | 0.067 |  |

APC: Antigen-presentation cell; HR: hazard ratio; OS: Overall survival; DFS: Disease-free survival; Ns: No significance
