# Supplementary material for: Identification of inhibitory immune checkpoints and relevant regulatory pathways in breast cancer stem cells
Source: Cancer Med. 2021 May 1;10(11):3794–807. doi: 10.1002/cam4.3902 (PMC8178503; doi:10.1002/cam4.3902)
Supplement: Supplementary file 10 — Table S2 [file CAM4-10-3794-s010.docx]

**Table S2 List of Wnt, TGF-β, and Hedgehog signalling-related CD200 & CD276 signatures**

| Wnt signalling-related CD200 & CD276 signature | TGF-β signalling-related  CD200 & CD276 signature | Hedgehog signalling-related  CD200 & CD276 signature |
| --- | --- | --- |
| CD200 | ACVRL1 | CD200 |
| CAMK2B | BMP2 | CD276 |
| CCND2 | BMPR2 | GAS1 |
| CD276 | CD200 | GLI1 |
| CREBBP | CD276 | GLI2 |
| DAAM1 | CDKN2B | GLI3 |
| DAAM2 | CREBBP | PTCH1 |
| EP300 | EP300 |  |
| FOSL1 | ID1 |  |
| FZD1 | ID4 |  |
| FZD4 | PITX2 |  |
| LEF1 | RBL1 |  |
| MAPK8 | ROCK1 |  |
| PLCB4 | TGFB3 |  |
| SFRP1 | TGFBR2 |  |
| TCF7L1 | THBS2 |  |
| WNT1 |  |  |
| WNT2 |  |  |
| WNT2B |  |  |
| WNT5A |  |  |
| WNT5B |  |  |
